# Problems associated with reconstitution, administration, and storage of antibiotic suspensions for pediatrics: a cross-sectional study in Nablus city, Palestine

**DOI:** 10.1186/s13104-015-1746-z

**Published:** 2015-12-09

**Authors:** Rowa’ J. Al-Ramahi, Abd Al Naser Zaid, Haya Anabousi

**Affiliations:** Department of Pharmacy, Faculty of Medicine and Health Sciences, An-Najah National University, P.O.Box 7, Nablus, Palestine

**Keywords:** Antibiotic, Suspension, Pediatrics, Palestine

## Abstract

**Background:**

The objective of this study was to evaluate the appropriateness of antibiotic suspensions use for pediatrics among Palestinian mothers’ including their reconstitution, dose administration, duration, and storage conditions.

**Methods:**

This study was a questionnaire based cross sectional descriptive study. It was conducted at a governmental primary healthcare center and a pediatric private clinic in Nablus city between January and March 2013. A sample of 400 mothers were met and asked to answer a face to face questionnaire.

**Results:**

The results showed that 347 (86.8 %) of mothers told that they read instructions, 311 (77.8 %) could understand manufacturers’ instructions, and 176 (44.0 %) of mothers asked pharmacists for advice when instructions were not well understood. In order to prepare antibiotic suspension, 302 (75.5 %) used boiled then cooled water, 192 (48.4 %) of mothers used a syringe to measure the needed amount of water, and 304 (76.6 %) added water in two steps, 392 (98.0 %) of mothers claimed that they shook the suspension bottle before use. Regarding dose administration, 313 (78.2 %) considered syringe as the most accurate tool for dose administration, most of mothers told that they gave drug dose with major meals when direction were to give three times daily, about use duration only 6 (1.5 %) mothers said that they used antibiotic suspension after 2 weeks, and 53 (6.5 %) gave left over antibiotic suspension to another child. One hundred seventy seven (44.2 %) mothers told they stored dry powder antibiotic in medicinal cabinet, while 226 (56.5 %) of them stored suspension in refrigerator.

**Conclusion:**

The results reflect a good level of correct practice. However, there is a room for improvement. The pharmacists are recommended to explain the correct directions, to supply a syringe with suitable calibration for dose administration, and to counsel parents about suitable storage condition, frequency of dosing and duration of use.

## Background

Pediatric infectious diseases either viral or bacterial remain a very common community health problem with an average occurrence of 6–8 times a year [1]. When infection is caused by bacteria then antibiotic is the drug of choice, most of newborns, infants, and preschool children receive antibiotic in the form of dry powder for reconstitution to suspension before administration [2]. Antibiotics prescribed for infants and young children are usually dispensed as oral suspensions because of children’s inability to swallow tablets or capsules, unavailability of certain antibiotics in a chewable tablet form and the discomfort, expense, and associated risk of antibiotic injections [3]. Appropriate use of antibiotic suspensions includes the correct reconstitution, concentration, dose administration, duration of treatment, and storage conditions.

Oral liquid medications usually come with a dose delivery device such as medication cups, droppers, calibrated spoons, and syringes [4]. Household spoons should not be used for delivery of medications as they are not accurate. In fact these spoons are usually available in different sizes. Syringes have many advantages; they are accurate even for small volumes, they are easy to use and to be cleaned. Regarding dosing cups dosing error are common with them, so as a general role they should not be used for doses less than 5 ml even if the cup has calibration less than 5 ml [5].

Storage conditions are also important, manufacturer instructions should be followed exactly, manufactures’ instructions recommend that some antibiotic suspensions need refrigerator, while for other antibiotic suspensions we need to avoid refrigerator.

Antibiotics are misused because many patients do not take them according to their doctor or pharmacist instructions. They may stop taking their antibiotics too soon, before their illness is completely cured. Some patients save unused medicine and take it later for another illness, or pass it to other ill family members or friends and some patients go to the pharmacy and take antibiotics as an over the counter drugs [6].

The aim of this study was to evaluate possible problems associated with antibiotic suspensions use among Palestinian mothers including their reconstitution, dose administration and storage conditions.

## Methods

This study was a questionnaire based cross sectional descriptive study. It was conducted at a governmental primary healthcare center and a pediatric private clinic in Nablus city between January and March 2013. A convenience sample of 400 randomly selected mothers was included. All mothers who were prescribed antibiotic suspensions for their children in the previous visit were asked to answer a face to face questionnaire until the required sample was met. The sample size was calculated based on Raosoft sample size calculator. Data collection tool was a questionnaire designed based on extensive literature review of similar studies [1, 6–8]. The questionnaire included information about sociodemographic characteristics of study subjects in addition to questions to evaluate appropriate reconstitution, administration and storage of antibiotic suspensions. The study protocol was authorized by An-Najah National University Institutional Review Board (IRB) and the Ministry of Health before initiation of the study. An oral consent was obtained from the participant also. To evaluate the mothers’ practice, it was compared with the manufactures’ instructions on drug box and package insert of every antibiotic used; the instructions of 22 trade names were reviewed. The practice was defined correct if it was according to these instructions. Statistical analysis was performed by using Statistical Package for Social Sciences (SPSS version 16.0). Means ± standard deviations were computed for continuous data. Frequencies (percentages) were calculated for categorical variables. Categorical variables were compared using Chi square. A p-value of less than 0.05 was considered to be statistically significant for all analyses.

## Results

### Socio-demographic characteristics

Among 450 mothers approached, 400 women accepted to participate in the study giving a response rate of 88.9 %. Women age was mainly between 20 and 30 years 258 (64.5 %), with a mean age of 28.8 ± 6.2 years, range (17–52 years), 307 (76.8 %) were from Nablus city, 236 (59.0 %) of them had a child in the age group 1–3 years. Most of participants had high school or university degree 39.8 and 47.8 % respectively, 70.8 % of mothers had medical insurance, 68.5 % of participants reported medium monthly income, and 79.2 % were not working (Table 1).

**Table 1:** Socio-demographic characteristics of the mothers

Characteristics	Frequency	Percentage
Mothers age		
Less than 20 years	13	3.2
Between 20–30 years	258	64.5
Between 31–40 years	115	28.8
Above 41 years	14	3.5
Monthly income		
Low	63	15.8
Medium	274	68.8
High	63	15.8
Educational level		
Primary school	6	1.5
Middle school	44	11.0
High school	159	39.8
Diploma/University degree and more	191	47.8
Living place		
City	307	76.8
Village	90	22.5
Camp	3	0.8
Working mother		
Yes	83	20.8
No	317	79.2
Child age		
Less than 1 years	63	15.8
Between 1–3 years	236	59.0
Between 3.1–5 years	64	16.0
Above 5 years	37	9.2

### Prescribed antibiotics and indications

During the study period the doctors were mainly visited due to throat infection (pharyngitis) 110 (27.5 %), bronchitis 110 (27.5 %), and otitis media 108 (27 %) (Table 2), and the most commonly prescribed antibiotics were amoxicillin in 161 (40.2 %) children, amoxicillin + clavulanic acid in 110 (12.8 %) children and azithromycin in 68 (17.0 %) cases as shown in (Table 3).

**Table 2:** Indications for used antibiotics

Indication	Frequency	Percentage
Throat infection (pharyngitis)	110	27.5
Bronchitis	110	27.5
Otitis media	108	27.0
Tonsillitis	45	11.2
Urinary tract infection	14	3.5
Dental infection	4	1.0
Pneumonia	4	1.0
Skin infection	3	0.8
Sinusitis	1	0.2
Trauma	1	0.2

**Table 3:** The most commonly prescribed antibiotics

Prescribed antibiotic	Frequency	Percentage
Amoxicillin	161	40.2
Amoxicillin + clavulanic acid	110	27.5
Azithromycin	68	17.0
Erythromycin	25	6.2
Cefalexin	15	3.8
Cefdinir	8	2.0
Cefuroxime	6	1.5
Trimethoprim + sulfamethoxazole	3	0.8
Penicillin V	3	0.8
Clarithromycin	1	0.2

### Reading and understanding the instructions

Among the 400 mothers, 347 (86.8 %) claimed that they read the manufacturer instructions either on the box or in the package insert, 311 (77.8 %) of them told that they could understand the instructions. In case of not understanding the instructions, 176 (44.0 %) of participants asked for help from pharmacists, while 41 (10.2 %) asked their doctors (Table 4).

**Table 4:** Summary of some mothers’ answers (N = 400)

Characteristics	Frequency	Percentage
Read instructions		
Yes	347	86.8
No	50	12.5
Understand instructions		
Yes	311	77.8
No	45	11.2
Source of advice		
Pharmacist	176	44.0
Doctor	41	10.2
Others	180	45.5
Water used		
Boiled then cooled tap water	302	75.5
Mineral water	52	13.0
Tap water directly	31	7.8
Distilled water	7	1.8
Prepared by pharmacists	5	1.2
Already reconstituted	3	0.8
Tool used to administer dose		
Syringe	313	78.2
Household teaspoon	57	14.3
Medicinal spoon	20	5.0
Medicinal cup	10	2.5

### Reconstitution of antibiotic suspensions

In order to reconstitute dry powder antibiotic, most mothers 302 (75.5 %) used boiled then cooled tap water, 52 (13.0 %) used mineral water, 31(7.8 %) used tap water directly, 7(1.8 %) used distilled water, and 5 (1.2 %) of drugs were prepared by pharmacists (Table 4). Fortunately, the correct practice was followed in 310 (77.8 %) cases (using boiled then cooled tap water by 302 and using distilled water which was practiced by seven mothers and one pharmacist).

**Fig. 1:**
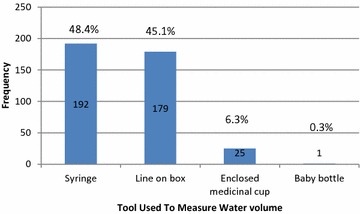
Tools used to measure water volume (N = 397)

Regarding the tool used to measure the volume of water for reconstitution, 192 mothers out of 397 who answered the question (48.4 %) used a syringe, while 179 (45.1 %) of participants used line on drug bottle, 25 (6.3 %) used enclosed medicinal cup, and 1 (0.3 %) used baby bottle (Fig. 1). According to the manufacturers’ instructions of medications used, the correct practice was followed by 344 (86.8 %) of mothers.

The water was added either in one step, two steps, or several steps. And the correct practice according to the instructions was done by 304 (76.6 %) of mothers.

### Administration of antibiotic doses

Most of the mothers 392 (98.0 %) claimed to shake the drug bottle before use which means that 98.0 % of mothers followed correct practice.

Among 400 mothers, 313 (78.2 %) of them considered syringe as the most accurate tool for drug administration, 57 (14.0 %), 20 (5.0 %), and 10 (2.5 %) of them considered household teaspoonful, medicinal spoonsful, and medicinal cup as the most accurate tool respectively, the correct practice according to related studies was practiced by 313 (78.2 %) of mothers.

**Fig. 2:**
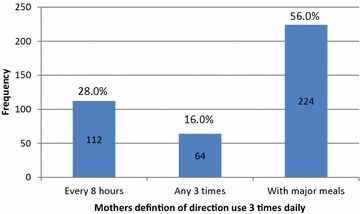
Mothers practice for direction “use 3 times daily”

The mothers were asked about their practice if the direction for antibiotic suspension is to use three times daily; 224 (56.0 %) of mothers told that they would administer the dose with major meals as shown in Fig. 2, the correct practice was followed by 112 (28.0 %) of mothers only.

Regarding the duration of administration; 394 (98.5 %) of mothers did not used suspension after 2 weeks of reconstitution, and 374 (93.5 %) of mothers did not use the leftover (remnant) for another child. Fortunately the correct practice according to manufacturer instructions was followed by 394 (98.5 %) and 374 (93.5 %) respectively.

### Storage conditions for antibiotic suspensions

Mothers were asked about the storage of dry powder; 177 (44.2 %) of mothers told that they stored dry powder antibiotic in medicinal cabinet, 72 (18.0 %) in kitchen, 56 (14.0 %) in refrigerator, 48 (12.0 %) in dining room, 22 (5.5 %) above refrigerator, and 25 (6.2 %) of mothers used antibiotic directly, and the correct practice (storing in medicinal cabinet, and using directly) was followed by 202 (50.5 %) of mothers.

**Fig. 3:**
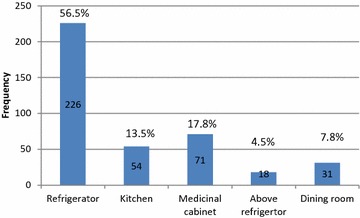
Storage condition of antibiotic suspension (N = 400)

Storage of suspension after reconstitution; 226 (56.5 %) of mothers told that they stored antibiotic suspension in refrigerator, 71 (17.8 %) in medicinal cabinet, 54 (13.5 %) in kitchen, 31 (7.8 %) in dining room, and 18 (4.5 %) above refrigerator (Fig. 3), and the correct practice was done by 200 (50.0 %) of mothers.

In this study, Chi square test showed no significant association between correct practice and any socio-demographic characteristics.

## Discussion

When infection is caused by bacteria then antibiotic is the drug of choice [1]. To achieve therapeutic effect and prevent treatment failure, antibiotic must be properly used. Suitable dosage forms for pediatric use are usually available in pharmacy [9]. Among these dosage forms dry antibiotic suspensions are the most commonly used [10]. Most antibiotics are water sensitive; accordingly they should be formulated as dry suspension for reconstitution [4]. The reconstitution requires addition of suitable pharmaceutical solvent which is water.

For optimal benefit from antibiotic suspension manufacturer instructions should be followed exactly. This study showed that 347 (86.8 %) of 400 mothers read the instructions either on box or package insert, and 311 (77.8 %) told that they understood the instructions. This is a good level of awareness and understanding compared to others Palestinian studies, a previous study showed that 100 (45.0 %) of 222 consumers reported that they always read information in the leaflet of the drug package [11], another showed that 51.7 % of 371 participants read the patient package insert [12]. It is recommended to make these instructions clear and complete as much as possible, and to encourage patients or caregivers to read instruction always.

Reconstitution of dry powder antibiotic depends on type of antibiotic and manufacturers’ instructions. Generally distilled or boiled then cooled tap water is used. First the bottle should be tabbed few times to loose the powder, then approximately half volume of water should be added, the bottle is shaken vigorously, the remaining of water should be added and shaken well. One exception is zinnat (cefuroxime) where the manufacturer recommends addition of water in one step. In this study 302 (75.5 %) of mothers used boiled then cooled tap water, 7 (1.8 %) of mothers used distilled water, this is a correct practice. While 52 (13.0 %) used mineral water, and 31 (7.8 %) used tap water directly, this is a wrong practice. As mineral water is usually rich in minerals, this may cause decomposition of drug and complexion reactions [13]. Among mothers, 304 (76.6 %) followed the correct practice in addition of water in two steps. Addition of water in one step makes it difficult to get the lumps out [4], while measuring volume of water several times increases the percentage of error in measured volume [14].

Regarding the volume of water required to reconstitute the powders, the correct practice in this study was followed by 344 (86.6 %) of mothers. In a previous Palestinian study, 3.5 % said the quantity of water that should be added to prepare suspension should not be a specific quantity, and 10.1 % could not decide or had no idea wither it should be specific or not [6]. It would be a good practice if pharmacists reconstitute antibiotics in pharmacy using suitable cylinders and suitable water.

Appropriate dose administration includes shaking the bottle before use, and using accurate delivery tool, and dose interval. In this study most of the mothers 392 (98.0 %) claimed to shake the drug bottle before use. In a study from Nigeria, among 107 mothers 24.4 % did not shake the bottle before measuring the dose volume [15]. In fact this wrong practice will lead to incomplete or wrong mixing which will result in missing the uniformity of doses. In general our results are excellent and reflect high level of awareness regarding this point.

According to this study 313 (78.2 %) of mothers considered syringe as the most accurate device for dose administration, while 57 (14.3 %) considered household teaspoonful as the most accurate device for dose administration. The correct practice was followed by most of the mothers. A Ghanaian study showed that 95.0 % of 97 respondents used household spoons in dosing oral liquid medications [7]. In another study majority of adult believed that oral syringe and dosing cup would measure an accurate dose [16]. Clinicians and pharmacists need to be aware that many people continue to use inaccurate devices for measuring liquid medication, such as household spoons. They should encourage the use of more accurate devices, particularly the oral dosing syringe. Clinicians should always consider the possibility of a medication dosing error when faced with an apparent treatment failure [17].

Regarding to dosing interval a high percentage of mothers 224 (56.0 %) assumed that “use three times daily” means to give the drugs with major meals and this is a wrong practice. As we know three times daily for antibiotics means to administer the medication every 8 h. Regarding to this point, the role of pharmacists is very important and they should write the proper instructions and confirm on them during dispensing to avoid antibiotic failure or toxicity if the dosing interval are not followed properly.

According to manufacturers’ directions antibiotic suspensions should be discarded after 10 days of reconstitution for antibiotics as amoxicillin, cefidinir, and cefuroxime, while amoxicillin–clavulanic acids, erythromycin, cephalexin should be discarded after 7 days, and after 5 days for azithromycin. This practice was followed by most mothers 394 (98.5 %). Regarding the use of left over suspensions, 26 (6.5 %) of mothers used left over antibiotic suspensions. Another study showed that 15.0 % of parents gave left over antibiotic suspension to their children which means that they keep antibiotics after reconstitution [1]. As it is well known, the medications should not be given to other individuals, this practice was followed by 374 (93.5 %) of mothers. A Malaysian study showed that 24.0 % of parents gave shared antibiotics [1], so our results reflect good awareness regarding rational use of antibiotics.

Storage conditions of drugs are important as drugs are chemicals that may react with external environment such as temperature, humidity, and light. This leads to changes in drugs concentration properties and therapeutic effects. Many people stored medications in kitchen or bathroom cabinet, that speeds up medication breakdown process; as in both conditions drugs may be exposed to humidity and high temperature. Instead medicinal cabinet should be placed in a cool and dry place, away from direct sun light, and out of reach from children. In this study a good percentage of mothers 177 (44.2 %) stored dry powder antibiotic in medicinal cabinet, and 226 (56.5 %) of them stored antibiotic suspension in refrigerator. In both cases around half of the mothers followed the correct practice. These results are close to results from another Palestinian study which showed that 40.6 % of drugs were stored in pharmacy cabinet; however, the same study showed that 70.0 % of the reconstituted suspensions were stored outside the refrigerator [18]. Regarding this point, more education and counseling is recommended because many people store medications in inappropriate places.

To achieve higher level of awareness and correct practice regarding antibiotic suspensions use, cooperation between drugs companies, pharmacists, and doctors is needed. Drugs companies are recommended to write instructions in a simple way, and may draw the directions on bottle, in addition to providing suitable dose administration devices. The prescribers should inform mothers the correct instructions also and confirm that antibiotic suspensions should not be shared between children. It is the responsibility of pharmacists to explain instructions to mothers and confirm on them by writing, and to provide mothers with syringes, also it is important to concentrate on duration of use and storage conditions.

The first limitation of this study is that the answers reported by the respondents cannot be validated and recall bias is possible, but this cannot be avoided in survey studies. Another limitation is the study was performed in one city only so it might not be representative to the practice in other places. However, these results can give a baseline data that can be useful in designing and implementing suitable educational programs and performing other related studies.

## Conclusion

The results reflect a good level of correct practice. However, there is a room for improvement. The pharmacists are recommended to explain the correct directions, to supply a syringe with suitable calibration for dose administration, and to counsel parents about suitable storage conditions, frequency of dosing and duration of use.
